# Naeso-san, a traditional herbal formula, attenuates HCl/ethanol-induced gastric injury via MAPK and NF-κB pathway modulation in mice

**DOI:** 10.3389/fphar.2025.1672854

**Published:** 2026-01-12

**Authors:** Suji Choi, In Gyoung Ju, Minji Lee, Seungmin Lee, Minsik Choi, Seong Hye Kim, Seong-Hoon Park, Hyangsook Lee, Young Pyo Jang, Eugene Huh, Myung Sook Oh

**Affiliations:** 1 Department of Formulae Pharmacology, College of Korean Medicine, Gachon University, Seongnam-si, Republic of Korea; 2 Division of Pharmacology, College of Korean Medicine, Semyung University, Jecheon-si, Republic of Korea; 3 Department of Oriental Pharmaceutical Science, College of Pharmacy, Kyung Hee University, Seoul, Republic of Korea; 4 Department of Biomedical and Pharmaceutical Sciences, Kyung Hee University, Seoul, Republic of Korea; 5 Division of Pharmacognosy, College of Pharmacy, Kyung Hee University, Seoul, Republic of Korea; 6 Genetic and Epigenetic Toxicology Research Group, Korea Institute of Toxicology, Daejeon, Republic of Korea; 7 Department of Medical Science of Meridian, College of Korean Medicine, Kyung Hee University, Seoul, Republic of Korea; 8 Acupuncture and Meridian Science Research Center, College of Korean Medicine, Kyung Hee University, Seoul, Republic of Korea; 9 Department of Integrated Drug Development and Natural Products, Graduate School, Kyung Hee University, Seoul, Republic of Korea

**Keywords:** anti-inflammatory effects, cytokine modulation, gastric injury, gastroprotection, MAPK signaling, naeso-san, NF-κB pathway, traditional botanical formulation

## Abstract

**Background:**

Gastric ulcers affect approximately 10% of the global population, while current acid-suppressive therapies have notable limitations including impaired digestion and long-term safety concerns. Naeso-san (NSS), a traditional botanical formulation, has shown promising gastroprotective effects, yet its precise molecular mechanisms remain incompletely understood. This study investigated the molecular pathways underlying its gastroprotective effects versus conventional therapies.

**Methods:**

We evaluated the gastroprotective effects of NSS (75, 300, 1200 mg/kg) in 7-week-old male ICR mice using a hydrochloric acid/ethanol (HCl/EtOH)-induced gastric injury model, with ranitidine (40 mg/kg) as positive control. Macroscopic damage scores were assessed, and molecular mechanisms including pro-inflammatory cytokines and mitogen-activated protein kinase (MAPK), protein kinase B (AKT), and nuclear factor-κB (NF-κB) signaling pathways were analyzed. *In vitro* studies using TNF-α-stimulated human gastric adenocarcinoma cell line (MKN45) gastric epithelial cells assessed inflammatory gene expression and cell viability.

**Results:**

NSS demonstrated dose-dependent gastroprotection with superior efficacy compared to ranitidine. While ranitidine effectively reduced macroscopic damage and TNF-α mRNA expression, it showed no significant effects on IL-1β expression or JNK, p38, AKT, and NF-κB signaling pathways. In contrast, NSS significantly suppressed pro-inflammatory cytokines and comprehensively inhibited multiple molecular pathways including MAPK, AKT, and NF-κB activation across all doses. *In vitro* studies confirmed dose-dependent suppression of TNF-α-induced inflammatory gene expression (IL-6, IL-8, IL-1β, COX-2) without cytotoxicity.

**Conclusion:**

NSS exhibits gastroprotective effects through multi-target anti-inflammatory mechanisms. These mechanistic advantages over conventional acid-suppressive therapies suggest NSS as a promising candidate for preclinical and translational studies evaluating its clinical applicability in inflammatory gastric conditions.

## 1 Introduction

Gastric ulcers affect approximately 10% of the global population with rising incidence. Notably, about 1% of patients face increased gastric cancer risk, prompting the World Health Organization to classify gastric ulcers as a precancerous condition (Yang et al., 2017; Liu et al., 2021). *Helicobacter pylori* infection represents a major pathogenic contributor, activating interleukin-6 (IL-6)/signal transducer and activator of transcription 3 (STAT3) signaling through reactive oxygen species-mediated IL-6 upregulation in gastric epithelial cells (Piao et al., 2016).

Gastritis pathogenesis involves complex inflammatory cascades regulated by key signaling pathways. Pro-inflammatory cytokines, including tumor necrosis factor-α (TNF-α), IL-6, and interleukin-1β (IL-1β), play crucial roles in gastric inflammation and tissue damage. These inflammatory responses are primarily mediated by mitogen-activated protein kinase (MAPK) and nuclear factor-κB (NF-κB) signaling pathways (Liu et al., 2021; Yeo et al., 2018). Downregulation of NF-κB signaling significantly reduces pro-inflammatory cytokine production (Yeo et al., 2018).

Conventional therapies rely on histamine-2 (H_2_) receptor antagonists and proton pump inhibitors (PPIs), which primarily alleviate symptoms through acid suppression. PPIs irreversibly inhibit H^+^/K^+^-ATPase in gastric parietal cells, resulting in sustained acid reduction (Shin and Kim, 2013). However, prolonged acid suppression compromises protein digestion and induces adverse effects such as headache, diarrhea, gynecomastia, hepatitis, and osteoporosis. Long-term PPI use has been associated with increased risks of renal, hepatic, and cardiovascular diseases, as well as dementia and infections (He et al., 2019; Freedberg et al., 2017; Yibirin et al., 2021).

These limitations have spurred interest in alternative gastroprotective strategies that enhance mucosal protection and promote regeneration (Yang et al., 2017). Various natural compounds—including *Inulae Flos* extract, sinapic acid, ponciretin, *Artemisia asiatica*, and *Pyrus ussuriensis* extracts—have demonstrated gastroprotective effects through modulation of multiple inflammatory pathways (Park et al., 2008; Kang and Kim, 2017; Yan et al., 2019; Kim et al., 2020; Boby et al., 2021; Raish et al., 2021). In parallel, recent integrative studies highlight that traditional Chinese medicine (TCM) formulas exert therapeutic benefits through multitarget, multilevel, and multi-pathway mechanisms, involving redox balance, inflammatory regulation, angiogenesis, mucosal protection, and modulation of gut microbiota and host metabolism. Despite these advantages, the precise molecular mechanisms underlying the actions of TCM formulations remain insufficiently defined, underscoring the need for targeted experimental validation (Gong et al., 2024; Wang et al., 2024). Despite these advantages, the precise molecular mechanisms underlying the actions of TCM formulations remain insufficiently defined, underscoring the need for targeted experimental validation.

Naeso-san (NSS; 內消散, Nèixiāosǎn in Chinese pinyin), a traditional herbal formulation documented in Wànbìng Huíchūn (萬病回春, ‘Complete Recovery from Myriad Diseases') by Gong Tingxian (龔廷賢, 1522-1619), has historically been used to treat gastrointestinal disorders including indigestion, food stagnation, vomiting, and abdominal distension (Kim and Yoon, 2008). Previous studies suggest that NSS exerts anti-inflammatory and mucosal protective effects by suppressing NF-κB p65, inducible nitric oxide synthase (iNOS), and cyclooxygenase-2 (COX-2) expression (Park and Baik, 2013). Additionally, NSS has been reported to dose-dependently inhibit pacemaker potentials in interstitial cells of Cajal via nitric oxide/cyclic guanosine monophosphate (NO/cGMP) signaling (Hong et al., 2014).

Despite these promising findings, the precise molecular mechanisms underlying NSS gastroprotection remain incompletely understood. This study evaluated the gastroprotective effects of NSS in a hydrochloric acid/ethanol (HCl/EtOH)-induced gastric injury model using mice, with ranitidine as positive control (P/C). We analyzed pro-inflammatory cytokine expression (IL-1β, TNF-α) and examined MAPK (c-Jun N-terminal kinase [JNK], p38), protein kinase B (AKT), and NF-κB signaling pathways. Additionally, *in vitro* studies using TNF-α-stimulated human gastric adenocarcinoma cell line (MKN45) gastric epithelial cells assessed inflammatory gene expression and cell viability.

## 2 Materials and methods

### 2.1 Preparation of NSS extract

NSS was prepared based on the standard formula specified in the Korean Pharmacopoeia for Herbal (Crude) Drugs Outside the Korean Pharmacopoeia (Ministry of Food and Drug Safety, 2024). The original formula comprises 11 herbal components in equal proportions (1.25 g each); however, Massa Medicata Fermentatawas excluded due to the difficulty in standardizing quality control for this fermented multi-ingredient preparation. The final NSS formulation consisted of 10 authenticated botanical drugs: *Citrus reticulata* Blanco, *Pinellia ternata* Breitenbach, *Poria cocos* Wolf, *Poncirus trifoliata* Rafinesque, *Crataegus pinnatifida* Bunge, *Amomum villosum* Loureiro var. *xanthioides* T. L. Wu et Senjen, *Cyperus rotundus* Linné, *Sparganium stoloniferum* Buchanan-Hamilton, *Curcuma phaeocaulis* Val., and *Zingiber officinale* Roscoe, each in equal proportions. These were decocted in distilled water (1:20, w/v) under reflux for 2 h and subsequently freeze-dried for 48 h to yield powdered extract (yield: 17.45%). A stock solution (20 mg/mL) was prepared in distilled water and diluted to appropriate concentrations for experiments. Details of the herbal composition are provided in Table 1.

**TABLE 1:** Composition of botanical drugs in Naeso-san.

Botanical drug	Voucher number from the Korea Ministry of food and drug safety	Dosage per single dose (g)	Ratio in Naeso-san
*Citrus reticulata* Blanco	CIRE2015	1.25	1
*Pinellia ternata* Breitenbach	PITE2013	1.25	1
*Poria cocos* wolf	POCO2016	1.25	1
*Poncirus trifoliata* rafinesque	POTR2008-1	1.25	1
*Crataegus pinnatifida* Bunge	CRPI2016	1.25	1
*Amomum villosum* loureiro var. *xanthioides* T. L. Wu et senjen	AMXA2016	1.25	1
*Cyperus rotundus* linné	CYRO2008	1.25	1
*Sparganium stoloniferum* Buchanan-Hamilton	SPST2010	1.25	1
*Curcuma phaeocaulis* val	CUPH2012	1.25	1
*Zingiber officinale* roscoe	ZIOF2016	1.25	1

### 2.2 High performance liquid chromatography (HPLC) analysis

Purified water obtained using the ABBOTA NEO system (UMC Science Co., Ltd., Ilsan, Republic of Korea) and methanol (Duksan Pure Chemicals Co., Ltd., Ansan, Republic of Korea) were employed to prepare sample and standard solutions for the quantification of compounds in the NSS extract. HPLC-grade acetonitrile and water (Fisher Scientific Korea Ltd., Seoul, Republic of Korea) and HPLC-grade acetic acid (Sigma-Aldrich Korea, Seoul, Republic of Korea) were used for mobile phase preparation. Reference standards of poncirin, naringin, and hesperidin (ChemFaces, Wuhan, China) were used for standard solutions preparation.

For the quantification of marker compounds in the NSS, 2.0 mg of the extract was dissolved in 1.0 mL of 50% methanol, sonicated for 15 min, and filtered through a 0.20 µm PVDF syringe filter (Whatman International Ltd., Maidstone, Kent, UK). The resulting solution was designated as the NSS test solution (NSTS).

Poncirin standard solutions (PSS) were prepared in triplicate by dissolving 0.5 mg of poncirin in 1.0 mL of 50% methanol, followed by serial dilution with 50% methanol to yield concentrations of 7.8125, 31.25, 62.5, 125, and 500 μg/mL. These five concentrations of PSS were analyzed to construct the poncirin calibration curve.

Mixed standard solutions were also prepared to determine the relative response factors (RRFs) between naringin and poncirin (RRF_Nar-Pon_), and between hesperidin and poncirin (RRF_Hes-Pon_). For the mixed standard solution of naringin with poncirin (MSS_Nar-Pon_), naringin was dissolved in 50% methanol at 250 μg/mL and combined with a poncirin standard solution of the same concentration at a 1:1 ratio. The mixed standard solution of hesperidin with poncirin (MSS_Hes-Pon_) was prepared in the same manner.

HPLC analysis of the NSTS, PSS, MSS_Nar-Pon_, and MSS_Hes-Pon_ was performed using a Waters HPLC e2695 system equipped with a photodiode array detector (PDA 2998; Waters Corp., Milford, MA, USA). Separation was achieved on a Luna® C18(2) column (5 μm, 100 Å, 250 × 4.6 mm; Phenomenex, Torrance, CA, USA). Chromatograms were recorded at 280 nm, corresponding to the λ max of the marker compounds. The injection volume was 10 μL, and the column and autosampler temperatures were maintained at 25 °C. The flow rate was set to 1.0 mL/min. The mobile phase consisted of acetonitrile containing 0.1% acetic acid (A) and water containing 0.1% acetic acid (B). The gradient elution of solvent A was programmed as follows: 0–10 min, 20%; 10–25 min, 20%–30%; 25–50 min, 30%–65%; 50–51 min, 65%–100%; 51–60 min 100% (column wash); followed by re-equilibration at 20% for 10 min.

### 2.3 Reagents and materials

Fetal bovine serum (Cat# 16000044) and phosphate-buffered saline (Cat# 70011044) were purchased from Gibco (Thermo Fisher Scientific, Waltham, MA, USA). RPMI-1640 medium (Cat# 1040-CV) was obtained from Corning (Corning, NY, USA). 3-(4,5-dimethylthiazol-2-yl)-2,5-diphenyl-2H-tetrazolium bromide (MTT) reagent (Cat# M1415.0001) was obtained from Duchefa Biochemie (Haarlem, Netherlands), and dimethyl sulfoxide (Cat# 35535S0350) from Junsei Chemical Co. (Tokyo, Japan). SYBR Green I Master Mix (Cat# QPK-201) was purchased from TOYOBO (Osaka, Japan). Nuclease-free water (TOYOBO, Osaka, Japan) was used without an assigned catalog number. The following primary antibodies were used for Western blotting: t-AKT (#9272), p-AKT (#9271), IL-1β (sc-32294; Santa Cruz), actin (sc-47778; Santa Cruz), p-JNK (#9251), t-JNK (#9252), p-p38 (#9211), t-p38 (#9212), p-NF-κB (#3033), t-NF-κB (#3034), and GAPDH (#5174). Quantitative PCR primers were purchased from Cosmogenetech (Seoul, Republic of Korea), and the primer sequences are listed in Table 2.

**TABLE 2:** Primer sequences for qRT-PCR.

Origin	Gene	Forward primer (5′→3′)	Reverse primer (5′→3′)
Mouse	*Tnf*	GAT​TAT​GGC​TCA​GGG​TCC​AA	GCT​CCA​GTG​AAT​TCG​GAA​AG
Mouse	*Il1b*	CCC​AAG​CAA​TAC​CCA​AAG​AA	GCT​TGT​GCT​CTG​CTT​GTG​AG
Mouse	*Gapdh*	TGA​ATA​CGG​CTA​CAG​CAA​CA	AGG​CCC​CTC​CTG​TTA​TTA​TG
Human	*IL6*	CCT​GAA​CCT​TCC​AAA​GAT​GGC	TTC​ACC​AGG​CAA​GTC​TCC​TCA
Human	*IL8*	ACT​GAG​AGT​GAT​TGA​GAG​TGG​AC	AAC​CCT​CTG​CAC​CCA​GTT​TTT​C
Human	*IL1B*	CCA​GGG​ACA​GGA​TAT​GGA​GCA	TTC​AAC​ACG​CAG​GAC​AGG​TAC​AG
Human	*COX2*	TTC​AAA​TGA​GAT​TGT​GGG​AAA​ATT​GCT	AGA​TCA​TCT​CTG​CCT​GAG​TAT​CTT
Human	*GAPDH*	GAA​GGT​GAA​GGT​CGG​AGT​C	GAA​GAT​GGT​GAT​GGG​ATT​TC

### 2.4 Animals and ethical approval

Male ICR mice (7 weeks old, weighing 25–30 g) were purchased from Daehan Biolink (Eumseong, Republic of Korea) and housed in standard cages (40 × 25 × 18 cm) under controlled conditions (23 °C ± 1 °C, 60% ± 10% humidity, 12-h light/dark cycle) with free access to food and water. After a 1-week acclimatization period, animals were randomly assigned to experimental groups (n = 6 per group) using computer-generated sequences. Mice were fasted for 24 h with water *ad libitum* before the experiment. To minimize the potential influence of coprophagy during fasting and ulcer induction, mice were transferred to freshly cleaned cages prior to fasting. Cage cleaning was also performed regularly during the experimental period to further reduce coprophagy. All procedures were approved by the Institutional Animal Care and Use Committee of Kyung Hee University (IACUC approval no.: KHSASP-23-479) and conducted in accordance with the NIH Guide for the Care and Use of Laboratory Animals (Eighth edition). This study did not involve human participants.

### 2.5 Gastric ulcer induction and treatment protocol

NSS was administered orally once daily for four consecutive days at doses of 75, 300, or 1200 mg/kg (Kim and Yoon, 2008; Park and Baik, 2013). Ranitidine hydrochloride (40 mg/kg; Sigma-Aldrich, St. Louis, MO, USA; Cat# R101-1G, CAS# 66357-59-3), a histamine H_2_ receptor antagonist, was used as P/C under the same regimen. On the final day, gastric injury was induced by oral administration of 60% ethanol containing 150 mM HCl (300 μL per mouse), 1 h after the final treatment. Mice were euthanized 1 h later by cervical dislocation, and stomachs were harvested for macroscopic and histological evaluation.

### 2.6 Gastric tissue evaluation

Stomachs were excised, opened along the greater curvature, and rinsed with phosphate-buffered saline. Macroscopic gastric lesions were photographed and analyzed using ImageJ software (National Institutes of Health, Bethesda, MD, USA) to calculate the ulcerated area as a percentage of the total gastric mucosal surface area.

For histological analysis, gastric tissues were fixed in 10% neutral-buffered formalin for 24 h, dehydrated, embedded in paraffin, and sectioned at 4-μm thickness. Sections were stained with hematoxylin and eosin according to standard protocols. Histological changes were assessed under an optical microscope (Olympus, Tokyo, Japan), and images were captured with a digital imaging system. Histological damage was semi-quantitatively scored based on epithelial loss, edema, and inflammatory cell infiltration by a blinded observer using predefined criteria.

### 2.7 Western blot analysis

Gastric tissues were homogenized in radioimmunoprecipitation assay (RIPA) buffer supplemented with protease and phosphatase inhibitors. Protein concentrations were determined using the Bradford assay. Equal amounts of protein (20 μg) were separated on 10% SDS–polyacrylamide gels and transferred to polyvinylidene difluoride membranes. After blocking with 5% skim milk in Tris-buffered saline with Tween 20 for 1 h, membranes were incubated overnight at 4 °C with primary antibodies against IL-1β, phosphorylated and total JNK, p38, AKT, and NF-κB (1:1000 dilution). Membranes were then incubated with horseradish peroxidase-conjugated secondary antibodies (1:5000) for 1 h at room temperature (22 °C ± 2 °C). Bands were visualized using an enhanced chemiluminescence kit and LAS-4000 Mini system (Fujifilm, Tokyo, Japan). Densitometric quantification was performed using MultiGauge software with standardized parameters including background subtraction from adjacent areas without protein signal. Only bands showing signal intensity ≥2-fold above background noise were included in quantitative analysis. All target protein signals were normalized to β-actin or GAPDH and expressed as percentage relative to the normal control (NOR) group (n = 6 per group).

### 2.8 Gene expression analysis by quantitative reverse transcription polymerase chain reaction

Total RNA was extracted using the RNeasy Mini Kit (QIAGEN, Valencia, CA, USA) and reverse transcribed with the PrimeScript RT reagent kit with gDNA Eraser (TaKaRa Bio Inc., Otsu, Japan). Quantitative PCR was conducted using BioFACT 2X real-time PCR Master Mix containing SYBR Green I and high ROX reference dye (BIOFACT, Daejeon, Republic of Korea). Cycling conditions were 95 °C for 10 min, followed by 40 cycles of 95 °C for 15 s and 60 °C for 60 s. Gene expression was quantified using the 2^−^ΔΔ^Ct^ method and normalized to GAPDH (n = 3 per group). Primer sequences are listed in Table 2.

### 2.9 Cell culture and inflammatory response assays

MKN45 human gastric adenocarcinoma cells (Korean Cell Line Bank, Seoul, Republic of Korea) were cultured in RPMI-1640 medium supplemented with 10% fetal bovine serum and 1% penicillin–streptomycin at 37 °C in a humidified 5% CO_2_ incubator. Cells at 80%–90% confluence were used for experiments.

For cell viability assays, cells (2 × 10^4^/well) were seeded in 96-well plates, incubated for 24 h, and treated with NSS (1, 10, or 100 μg/mL), with or without TNF-α (40 ng/mL), for 24 h. After treatment, MTT (1 mg/mL) was added and incubated for 4 h. Formazan crystals were dissolved in DMSO, and absorbance was measured at 540 nm using a microplate reader (SpectraMax ABS, Molecular Devices, USA). Viability was expressed as a percentage relative to untreated controls (n = 5 per group).

For gene expression analysis, cells (4 × 10^5^/well) were seeded in 6-well plates and treated with NSS (1, 10, or 100 μg/mL) and TNF-α (40 ng/mL) for 24 h. Cells were washed, centrifuged at 3000 rpm for 5 min at 4 °C, and subjected to RNA extraction and qRT-PCR (n = 3 per group).

### 2.10 Statistical analysis

All data are presented as mean ± standard error of the mean. Statistical comparisons were performed using one-way analysis of variance followed by Dunnett’s *post hoc* test in GraphPad Prism version 8.0 (GraphPad Software, San Diego, CA, USA). Data normality was assessed using the Shapiro–Wilk test. A p-value of <0.05 was considered statistically significant.

## 3 Results

### 3.1 Standardization of NSS

**FIGURE 1:**
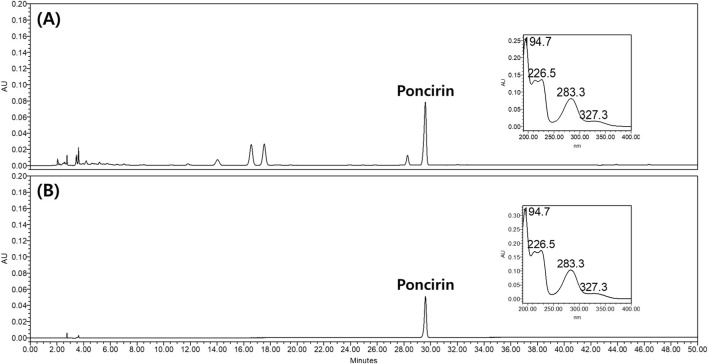
Chromatograms of the NSTS **(A)** and PSS **(B)** monitored at UV 280 nm, with UV spectra of the poncirin peak detected from each chromatogram.

To standardize NSS, we quantified the poncirin, naringin, and hesperidin using HPLC analysis. The poncirin content in the NSTS was quantified using the PSS as a reference. Poncirin was identified at a retention time of 29.6 min in both the NSTS and PSS chromatograms, with identical UV spectra (Figure 1). The standard calibration curve for poncirin exhibited excellent linearity, with a correlation coefficient (R^2^ = 0.9992) (Supplementary Figure S1). Based on this calibration curve, the concentration of poncirin in the NSTS were determined to be 53.31 μg/mL, corresponding to 0.027 mg per mg of the NSS.

In the NSTS chromatogram, naringin and hesperidin were detected at retention times of 16.6 and 17.5 min, respectively, without interference from adjacent peaks. Their UV spectra were consistent with those of MSS_Nar-Pon_ and MSS_Hes-Pon_, confirming peak identities (Supplementary Figure S2). Quantification of naringin and hesperidin was performed using the relative response factors (RRFs) between naringin and poncirin (RRF_Nar-Pon_), and between hesperidin and poncirin (RRF_Hes-Pon_). Based on the analysis of MSS_Nar-Pon_ and MSS_Hes-Pon_, the calculated RRF_Nar-Pon_ and RRF_Hes-Pon_ values were 0.836 and 0.038, respectively (Equation 1).

$${RRF}_{X-Pon}=\frac{\left(X\;peak\;area\;of\;{MSS}_{X-Pon}\right)÷\left(X\;concentration\;of\;{MSS}_{X-Pon}\right)}{\left(Poncirin\;peak\;area\;of\;{MSS}_{X-Pon}\right)÷\left(Poncirin\;concentration\;of\;{MSS}_{X-Pon}\right)}\;where\;X=naringin\;or\;hesperidin$$ (1)

The concentrations of naringin and hesperidin in the NSTS were then determined using Equation 2, based on the RRFs and the peak areas of poncirin, naringin, and hesperidin in the NSTS chromatogram.

$$X\text{ Concentration in the NSTS}=\frac{\left(X\;peak\;area\;in\;the\;NSTS\right)}{\left(Poncirin\;peak\;area\;in\;the\;NSTS\right)}\times \frac{\left(Poncirin\;concentration\;in\;the\;NSTS\right)}{{RRF}_{X-Pon}}\;where\;X=naringin\;or\;hesperidin$$ (2)

The calculated concentrations of naringin and hesperidin were 26.7 μg/mL and 601.2 μg/mL, respectively. Finally, the contents per 1 mg of the NSS were 0.027 mg for poncirin, 0.013 mg for naringin, and 0.30 mg for hesperidin.

### 3.2 Gastroprotective effects of NSS on macroscopic and histological gastric damage

**FIGURE 2:**
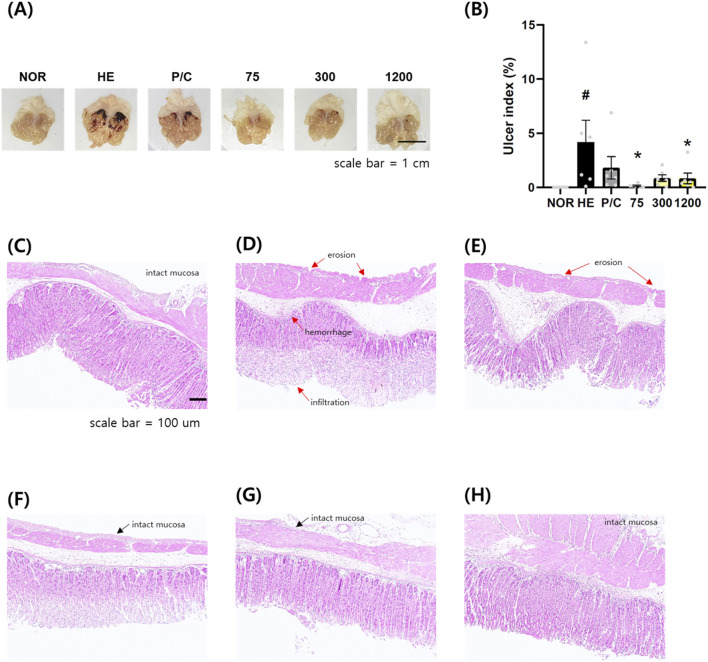
NSS mitigates macroscopic and histological gastric damage in HCl/ethanol-induced gastric injury. **(A)** Representative images were obtained to show the extent of gastric mucosal injury in each group (scale bar = 1 cm). **(B)** The ulcer index was calculated based on macroscopic lesions. **(C–H)** Histological sections stained with hematoxylin and eosin (H&E) (scale bar = 100 µm) illustrate changes in mucosal integrity, including epithelial erosion and inflammatory infiltration. Red arrows indicate mucosal erosion, hemorrhage, or inflammatory infiltration; black arrows indicate preserved mucosa. Data are presented as mean ± SEM (n = 6). *p < 0.05 vs. HE; #p < 0.05 vs. NOR. Abbreviations: NOR, normal control; HE, HCl/ethanol; P/C, ranitidine; NSS, Naeso-san. Individual data points are shown for each experimental group.

The gastroprotective effects of NSS were evaluated in an HCl/EtOH-induced gastric injury model. Mice in the HCl/EtOH group showed extensive hemorrhagic lesions and severe mucosal damage, while the NOR group maintained normal gastric morphology. Oral administration of NSS at 75, 300, and 1200 mg/kg visibly reduced gross gastric lesions in a dose-dependent manner, similar to the P/C group treated with ranitidine (40 mg/kg) (Figure 2A). Quantification of ulcerated areas demonstrated that NSS at 75 and 1200 mg/kg significantly reduced ulcer scores compared with the HCl/EtOH group, whereas the 300 mg/kg group showed a non-significant decreasing trend (Figure 2B).

Histological analysis with hematoxylin and eosin staining revealed prominent epithelial erosion, submucosal edema, and inflammatory infiltration in the HCl/EtOH group. These pathological features were substantially ameliorated in the P/C group and NSS-treated groups, with preservation of mucosal integrity and reduced inflammation, particularly at higher NSS doses (Figures 2C–H).

### 3.3 Anti-inflammatory effect of NSS through IL-1β and TNF-α modulation in HE-Induced gastric tissues

**FIGURE 3:**
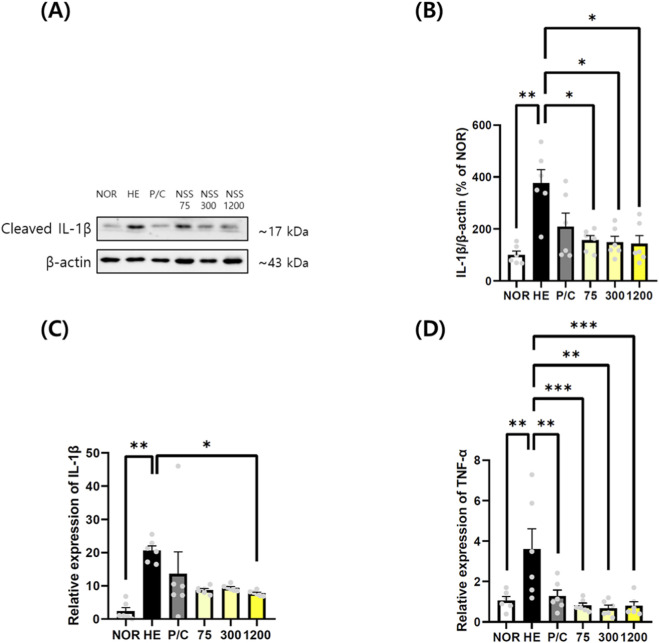
NSS downregulates IL-1β and TNF-α expression in HCl/ethanol-induced gastric tissues. **(A)** Western blotting was performed to evaluate cleaved IL-1β protein levels. (β-actin was used as a loading control). **(B)** Relative protein levels of cleaved IL-1β were quantified and normalized to β-actin. **(C)** Relative mRNA expression of IL-1β was measured using quantitative RT-qPCR. **(D)** Relative mRNA expression of TNF-α was also determined by RT-qPCR. Representative cropped blots are shown; uncropped full-length blots are provided in Supplementary Figure S3. Data are shown as mean ± SEM (n = 6). *p < 0.05, **p < 0.01, ***p < 0.001 vs. the group indicated (HE or NOR). Abbreviations: NOR, normal control; HE, HCl/ethanol; P/C, ranitidine; NSS, Naeso-san. Individual data points are shown for each experimental group.

To evaluate the anti-inflammatory effects of NSS, IL-1β and TNF-α levels were measured in gastric tissues. Western blot analysis showed significantly elevated cleaved IL-1β protein in the HCl/EtOH group (376.50% ± 51.19%) compared with the NOR group (100.00% ± 14.53%) (p < 0.01). NSS treatment significantly reduced IL-1β expression to 156.90% ± 17.13% (p < 0.05), 148.50% ± 23.09% (p < 0.05), and 143.20% ± 31.31% (p < 0.05) at 75, 300, and 1200 mg/kg, respectively (Figures 3A,B; Supplementary Figure S3). IL-1β mRNA expression was markedly elevated in the HCl/EtOH group (20.68 ± 2.17-fold) compared with the NOR group (2.61 ± 0.46-fold) (p < 0.01). NSS reduced IL-1β transcript levels, with significant suppression observed at 1200 mg/kg (6.60 ± 1.24-fold) (p < 0.05) (Figure 3C). TNF-α mRNA levels were also significantly increased in the HCl/EtOH group (3.61 ± 0.44-fold) relative to the NOR group (1.00 ± 0.19-fold) (p < 0.01). All NSS treatment groups showed statistically significant reductions: 0.69 ± 0.18-fold (p < 0.001), 1.00 ± 0.24-fold (p < 0.01), and 0.64 ± 0.15-fold (p < 0.001) at 75, 300, and 1200 mg/kg, respectively (Figure 3D).

### 3.4 Inhibitory effect of NSS on phosphorylation of JNK and p38 in HE-Induced gastric tissues

**FIGURE 4:**
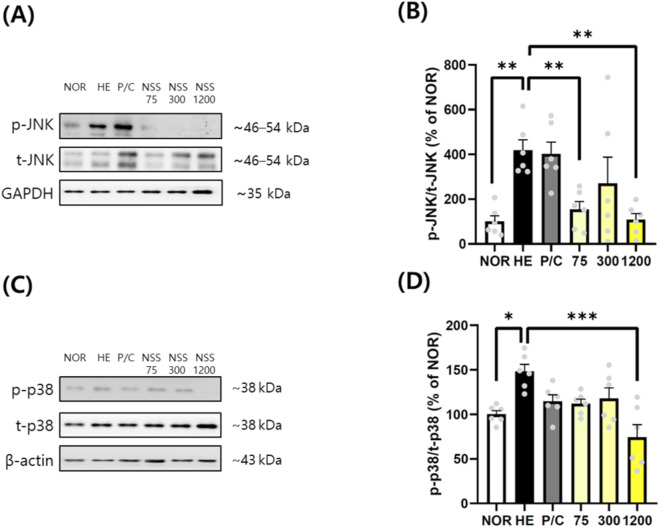
NSS inhibits phosphorylation of JNK and p38 in gastric tissues subjected to HCl/ethanol-induced injury. **(A)** Western blot analysis was conducted to assess the levels of phosphorylated and total JNK. **(B)** Relative protein expression of ratio of phosphorylated to total JNK (p-JNK/t-JNK) was calculated. **(C)** Western blotting was also used to evaluate phosphorylated and total p38. **(D)** Relative protein expression of ratio of phosphorylated to total p38 (p-p38/t-p38) was determined. Representative cropped blots are shown; uncropped full-length blots are provided in Supplementary Figure S4. GAPDH and β-actin were used as loading controls. Data are expressed as mean ± SEM (n = 6). *p < 0.05, **p < 0.01, ***p < 0.001 vs. the group indicated (HE or NOR). Abbreviations: NOR, normal control; HE, HCl/ethanol; P/C, ranitidine; NSS, Naeso-san. Individual data points are shown for each experimental group.

The impact of NSS on MAPK signaling was assessed via JNK and p38 phosphorylation levels. The HCl/EtOH group showed elevated phospho-JNK (418.20% ± 46.87%) compared with the NOR group (100.00% ± 25.48%) (p < 0.01). NSS at 75 and 1200 mg/kg significantly decreased JNK phosphorylation to 154.30% ± 34.67% (p < 0.01) and 108.10% ± 27.42% (p < 0.01), respectively (Figures 4A,B). Similarly, the phospho-p38/t-p38 ratio increased to 148.10% ± 7.93% in the HCl/EtOH group compared with 100.00% ± 4.04% in the NOR group (p < 0.05). NSS at 1200 mg/kg significantly reduced p38 phosphorylation to 74.29% ± 14.17% (p < 0.001) (Figures 4C,D; Supplementary Figure S4).

### 3.5 Effects of NSS on AKT and NF-κB phosphorylation in HE-Induced gastric tissues

**FIGURE 5:**
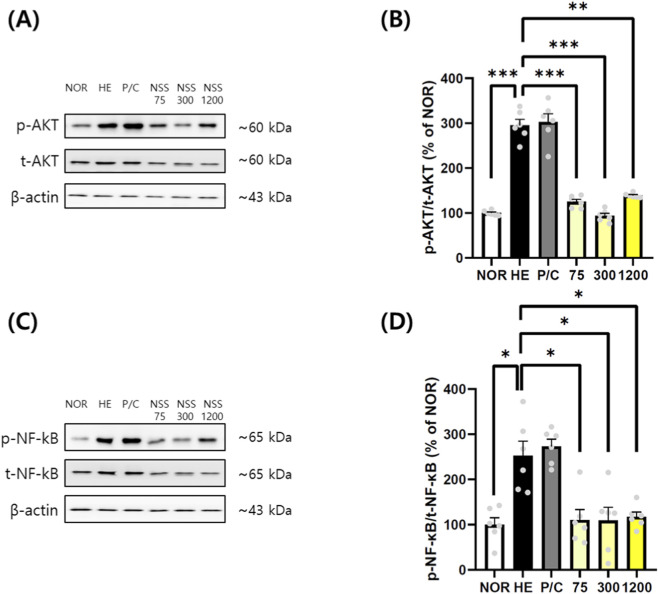
NSS suppresses AKT and NF-κB phosphorylation in gastric tissues following HCl/ethanol-induced injury. **(A)** Western blotting was performed to detect phosphorylated and total AKT. **(B)** Relative protein expression of ratio of p-AKT/t-AKT was quantified. **(C)** Levels of phosphorylated and total NF-κB were determined by Western blotting. **(D)** Relative protein expression of ratio of p-NF-κB/t-NF-κB was calculated. Representative cropped blots are shown; uncropped full-length blots are provided in Supplementary Figure S5 β-actin was used as a loading control. Data are presented as mean ± SEM (n = 6). *p < 0.05, **p < 0.01, ***p < 0.001 vs. the group indicated (HE or NOR). Abbreviations: NOR, normal control; HE, HCl/ethanol; P/C, ranitidine; NSS, Naeso-san. Individual data points are shown for each experimental group.

Phosphorylation of AKT was markedly increased in the HCl/EtOH group (295.30% ± 13.26%) compared with the NOR group (100.00% ± 2.30%) (p < 0.001). NSS at all tested doses significantly attenuated AKT phosphorylation: 124.40% ± 12.55% (p < 0.001), 97.56% ± 6.75% (p < 0.001), and 127.30% ± 15.53% (p < 0.01) at 75, 300, and 1200 mg/kg, respectively (Figures 5A,B). NF-κB activation was similarly increased in the HCl/EtOH group (252.80% ± 32.02%) relative to the NOR group (100.00% ± 15.58%) (p < 0.05). NSS significantly reduced NF-κB phosphorylation: 110.70% ± 29.62% (p < 0.05), 105.00% ± 20.41% (p < 0.05), and 109.40% ± 8.77% (p < 0.05) at 75, 300, and 1200 mg/kg, respectively (Figures 5C,D; Supplementary Figure S5).

### 3.6 *In Vitro* anti-inflammatory effects of NSS in TNF-α-stimulated gastric epithelial cells

**FIGURE 6:**
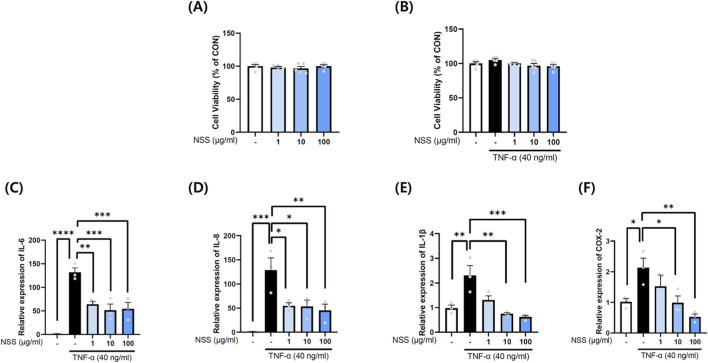
NSS preserves cell viability and reduces cytokine expression in TNF-α-stimulated MKN45 human gastric adenocarcinoma cells. **(A)** Cell viability was evaluated after treatment with various concentrations of NSS. **(B)** Cell viability was also assessed following TNF-α stimulation with or without NSS co-treatment. **(C–F)** Relative mRNA expression levels of IL-6, IL-8, IL-1β and COX-2 were measured using RT-qPCR. Data are expressed as mean ± SEM (cell viability: n = 5; gene expression: n = 3). *p < 0.05, **p < 0.01, ***p < 0.001, ****p < 0.0001 vs. the group indicated (control or TNF-α). Abbreviation: NSS, Naeso-san. Individual data points are shown for each experimental group.

To validate *in vivo* findings, MKN45 cells stimulated with TNF-α were treated with NSS. NSS at concentrations of 1, 10, and 100 μg/mL showed no cytotoxicity (viability: 97.88% ± 1.92%, 96.51% ± 3.14%, and 99.99% ± 2.17%, respectively) (Figures 6A,B). TNF-α significantly increased IL-6 expression (131.70% ± 9.67%) compared with control cells. NSS dose-dependently reduced IL-6 to 63.80% ± 6.62% (p < 0.01), 51.30% ± 13.15% (p < 0.001), and 54.44% ± 13.28% (p < 0.001) at 1, 10, and 100 μg/mL, respectively (Figure 6C). IL-8 expression followed a similar trend, increasing to 128.70% ± 25.20% with TNF-α and decreasing to 54.63% ± 6.16% (p < 0.05), 53.01% ± 13.36% (p < 0.05), and 44.89% ± 13.27% (p < 0.01) at 1, 10, and 100 μg/mL NSS, respectively (Figure 6D). IL-1β mRNA levels increased to 2.73 ± 0.51-fold with TNF-α and were significantly reduced to 0.75 ± 0.04-fold (p < 0.01) and 0.62 ± 0.07-fold (p < 0.001) with 10 and 100 μg/mL NSS, respectively, with no significant effect at 1 μg/mL (Figure 6E). COX-2 mRNA levels increased to 2.13 ± 0.31-fold with TNF-α and were significantly reduced to 0.98 ± 0.23-fold (p < 0.05) and 0.52 ± 0.09-fold (p < 0.01) with 10 and 100 μg/mL NSS, respectively (Figure 6F).

## 4 Discussion

**FIGURE 7:**
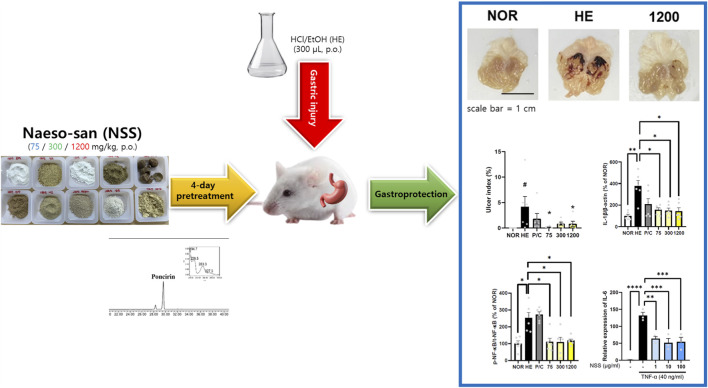
Graphical summary of the experimental workflow and major findings of NSS. The diagram illustrates the preparation of Naeso-san (NSS) with HPLC profiling (poncirin as a marker metabolite), the HCl/EtOH-induced gastric injury model in mice with 4-day pretreatment of NSS (75, 300, 1200 mg/kg), and representative outcomes including gross gastric images, ulcer index quantification, and molecular analyses. NSS suppressed pro-inflammatory mediators such as IL-1β, NF-κB phosphorylation, and IL-6 expression, thereby conferring gastroprotection through multi-target anti-inflammatory mechanisms.

NSS confers gastroprotective effects by inhibiting multiple inflammatory pathways in HCl/ethanol-induced acute gastric injury. This occurred via suppression of pro-inflammatory cytokines, MAPK signaling inhibition, and modulation of AKT/NF-κB activation. These effects occurred without cytotoxicity in gastric epithelial cells, suggesting a favorable therapeutic window. NSS thus offers a mechanistically distinct approach by targeting inflammation rather than merely suppressing acid (Figure 7).

In gastric injury models, the MAPK pathway—comprising JNK, p38, and ERK—plays a central role in transducing oxidative and inflammatory stress signals into cellular responses including apoptosis and cytokine production. JNK and p38 are particularly activated by reactive oxygen species and pro-inflammatory stimuli, leading to amplification of mucosal damage through downstream transcription factor activation. NF-κB serves as a master regulator of inflammatory gene expression, controlling the transcription of cytokines (TNF-α, IL-1β, IL-6), adhesion molecules, and enzymes such as COX-2. In HCl/ethanol-induced gastric injury, NF-κB activation has been shown to exacerbate mucosal inflammation and delay healing. Recent studies on gastroprotective natural products have demonstrated that inhibition of MAPK and NF-κB pathways correlates with reduced gastric damage (Liu et al., 2021). NF-κB inhibition has also been reported with sinapic acid (Raish et al., 2021) and Artemisia extracts (Yeo et al., 2018) in ethanol-induced gastric injury models.

A notable finding was the non-linear dose-response of AKT phosphorylation, with intermediate doses showing maximal inhibition. This biphasic pattern implies complex pharmacodynamic interactions such as receptor saturation, pathway crosstalk (e.g., PI3K/AKT and MAPK), or differences in phytochemical bioavailability (Shaik, 2024; Yadav et al., 2024). These dynamics underscore the need for dose-finding studies rather than assuming linearity. NSS’s multi-metabolite nature may produce synergistic effects at specific concentration ratios. Additionally, NSS at the lowest dose (75 mg/kg) demonstrated comparable or even superior gastroprotective effects to higher doses in certain parameters, particularly evident in the ulcer index measurements. This non-monotonic dose-response relationship is characteristic of multi-metabolite herbal formulations, where optimal biological effects may occur at specific concentration ranges rather than following linear dose-dependency.

NSS offers advantages over standard therapies such as PPIs and H_2_ antagonists, which, despite effective acid suppression, pose long-term risks (renal, hepatic, cardiovascular, infections) (Yibirin et al., 2021). Prolonged acid suppression impairs digestion and nutrient absorption and may paradoxically increase gastric cancer risk via atrophic gastritis. While ranitidine reduced gastric damage and TNF-α mRNA expression, it failed to affect IL-1β or inhibit JNK, p38, AKT, or NF-κB activation. This suggests H2 antagonism offers limited anti-inflammatory benefit. This interpretation is further supported by findings from another study conducted in the same HCl/ethanol-induced acute gastric injury model (Kim et al., 2021). In that study, ranitidine produced clear macroscopic gastroprotection but exhibited only limited suppression of intracellular inflammatory pathways, including NF-κB, COX-2 and iNOS, whereas the test substance demonstrated markedly greater anti-inflammatory efficacy. These results indicate that, in acute chemical injury models, H_2_-receptor antagonists do not consistently modulate cytokine-driven inflammatory signaling despite reducing gross ulceration. Thus, the absence of significant reductions in IL-1β and related signaling markers in the ranitidine group of our study is consistent with the model-dependent inflammatory kinetics and reflects a recognized limitation of ranitidine rather than an experimental inconsistency. In contrast, NSS exhibited broad suppression of inflammatory mediators across all tested doses. Notably, NF-κB inhibition—a key regulator of inflammatory gene transcription—highlights its therapeutic relevance in gastric inflammation and carcinogenesis (Kim et al., 2021; Bahrami et al., 2024). While we demonstrated significant TNF-α mRNA suppression (Figure 3D), protein-level confirmation would strengthen these findings. However, TNF-α protein quantification in gastric tissue presents technical challenges due to rapid secretion into circulation and local proteolytic degradation. The consistent suppression of IL-1β protein (Figures 3A,B), downstream inflammatory mediators (IL-6, IL-8 and COX-2; Figure 6), and multiple signaling pathways (Figures 4, 5) provides convergent evidence for comprehensive anti-inflammatory effects of NSS at both transcriptional and translational levels.

These findings align with emerging strategies emphasizing mucosal defense enhancement over acid neutralization, as seen in phytochemicals like sulforaphane targeting NF-κB and MAPK (Fernandes et al., 2024; Gan et al., 2023). Simultaneous inhibition of JNK/p38, AKT, and NF-κB reflects network-level targeting, consistent with the phytochemical complexity of NSS (e.g., flavonoids, sesquiterpenes, essential oils) (Kim and Yoon, 2008; Múnera-Rodríguez et al., 2024). Previous research showed NSS modulates pacemaker potentials in interstitial cells of Cajal via NO/cGMP and ATP-sensitive K^+^ channels (Hong et al., 2014), suggesting broader applicability to functional GI disorders. The anti-inflammatory effects observed may also benefit conditions such as inflammatory bowel disease, dyspepsia, GERD, and H. pylori-associated gastritis (Múnera-Rodríguez et al., 2024; Yu et al., 2022; Che et al., 2024; Han et al., 2024).

For clinical translation, dosage and safety must be clarified. Human-equivalent doses (75–1200 mg/kg in mice) yield 420–6790 mg/day for a 70 kg adult (Landrum and Riniker, 2024; FDA, 2005; Reagan-Shaw et al., 2008), within traditional decoction ranges (Yadav et al., 2024; Marks et al., 2018). Importantly, no cytotoxicity was observed up to the highest tested dose, supporting potential for chronic use. Nonetheless, safety evaluations are needed, particularly for interactions with common medications (e.g., anticoagulants, corticosteroids, NSAIDs) that may exacerbate gastric risk (Ahmad et al., 2024).

Limitations include use of an acute injury model, which may not reflect chronic gastritis (Lanas and Chan, 2017; Mizui and Doteuchi, 1983). Short-term exposure does not inform on durability, tolerance, or long-term safety. *In vitro* findings, though supportive, were limited to the MKN45 gastric adenocarcinoma cell line. Although MKN45 cells have been widely used for studying TNF-α-induced inflammatory signaling including NF-κB pathway activation (Cho et al., 2023), as a cancer-derived cell line, they may exhibit differences from normal gastric epithelial cells, including altered proliferation rates, constitutive activation of certain oncogenic pathways, and potentially different baseline inflammatory responses. Future studies using primary gastric epithelial cells or immortalized normal gastric cell lines such as GES-1 would further strengthen these findings and enhance clinical translatability (Ueda et al., 2003; Newman and Cragg, 2020).

## 5 Conclusion

NSS demonstrated gastroprotective effects against HCl/ethanol-induced gastric injury by comprehensively suppressing pro-inflammatory cytokines (IL-1β, TNF-α, IL-6 and IL-8) and modulating multiple signaling cascades including MAPK (JNK and p38), AKT and NF-κB pathways. These multi-target anti-inflammatory mechanisms, achieved through mechanisms distinct from acid suppression and without causing cytotoxicity, distinguish NSS from conventional acid-suppressive therapies and support its potential as a mechanistically distinct therapeutic candidate for inflammatory gastric conditions.

## Data Availability

The raw data supporting the conclusions of this article will be made available by the authors, without undue reservation.
