# Supplementary material for: Naeso-san, a traditional herbal formula, attenuates HCl/ethanol-induced gastric injury via MAPK and NF-κB pathway modulation in mice
Source: Front Pharmacol. 2026 Jan 12;16:1672854. doi: 10.3389/fphar.2025.1672854 (PMC12833003; doi:10.3389/fphar.2025.1672854)
Supplement: Supplementary file 1 [file Supplementaryfile1.docx]

**Supplementary Material**

The Supplementary Material for this article can be found online.


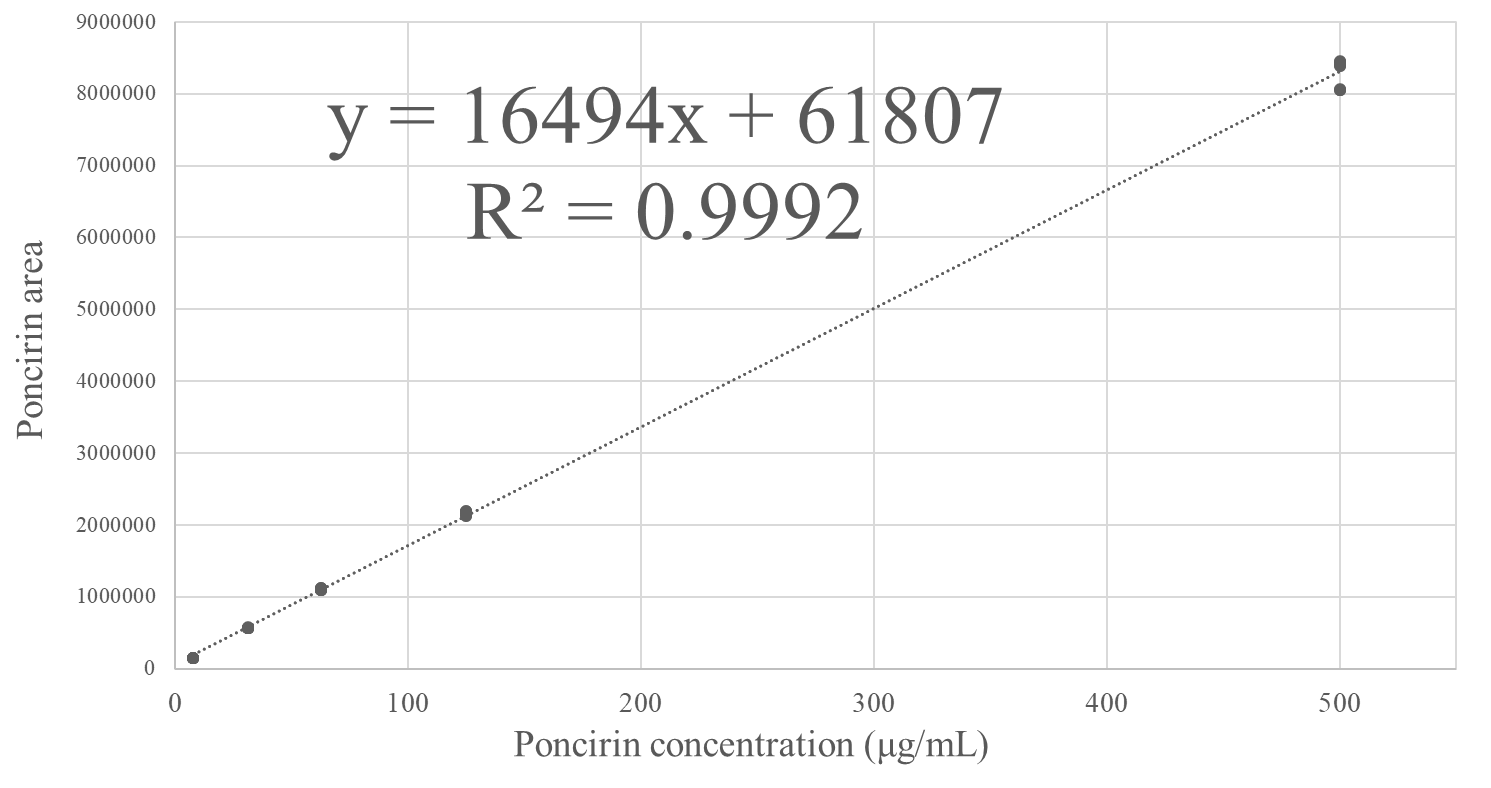


Supplementary Figure S1. Calibration curve of poncirin used for the standardization of NSS. Linear regression equation and correlation coefficient (R² = 0.9992) are shown.


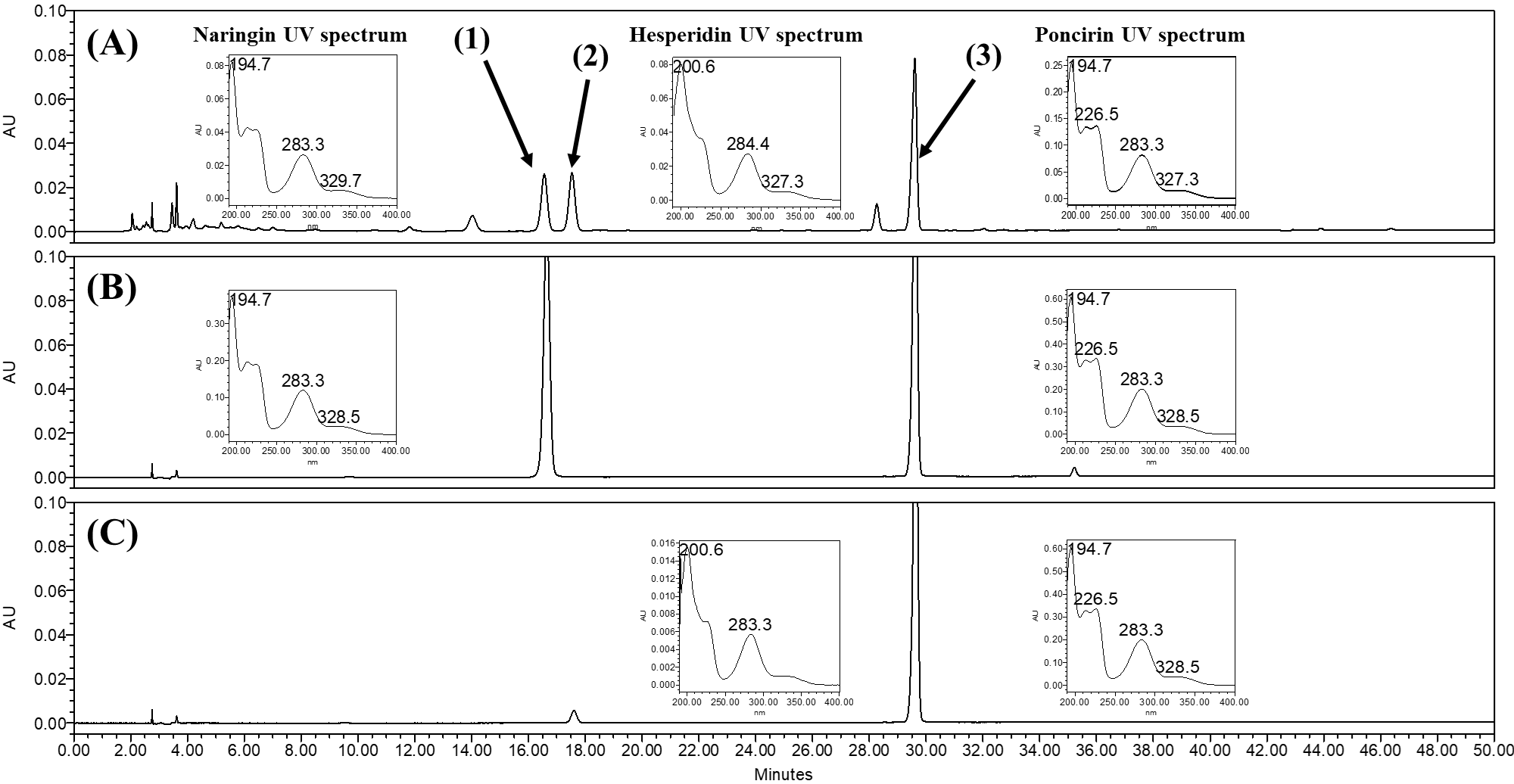


Supplementary Figure S2. UV spectra and chromatograms of marker metabolites in NSS. (A) Naringin, hesperidin, and poncirin in mixed standard solution. (B) Naringin with poncirin. (C) Hesperidin with poncirin. UV spectra confirm peak identities.


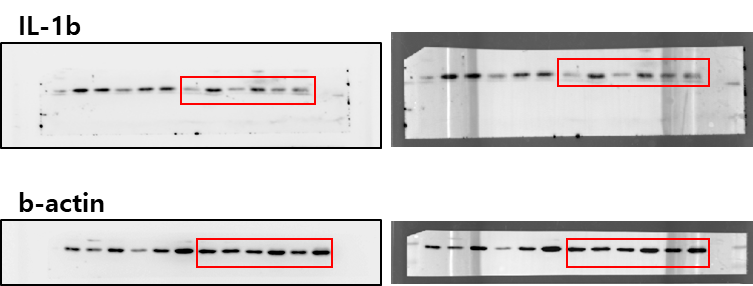


Supplementary Figure S3. Uncropped full-length Western blot membranes corresponding to Figure 3 (IL-1β). Red boxes indicate cropped regions shown in the main figure. β-actin was used as a loading control.


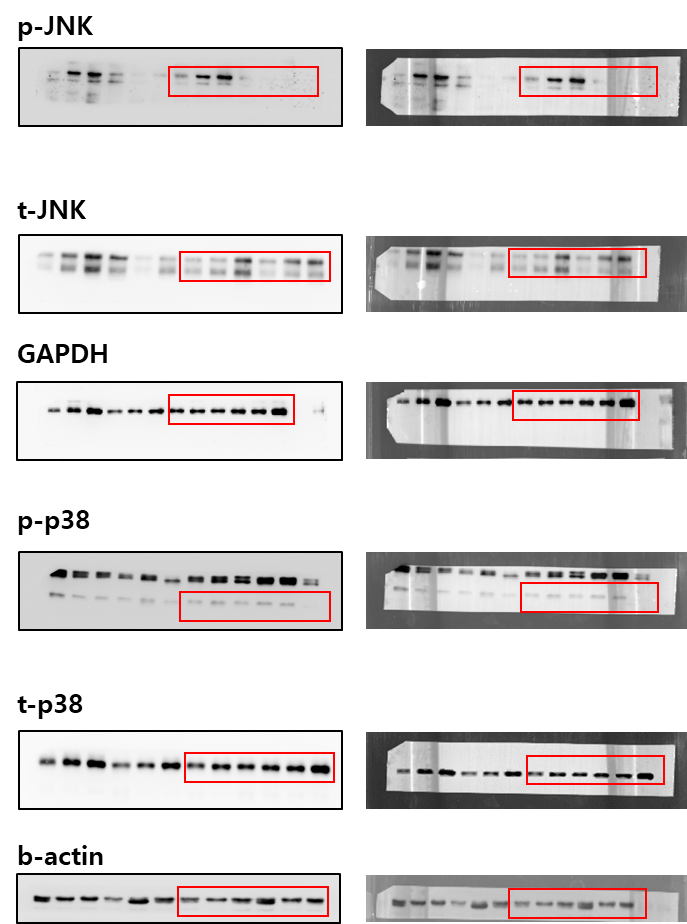


Supplementary Figure S4. Uncropped full-length Western blot membranes corresponding to Figure 4 (p-JNK, t-JNK, p-p38, t-p38). Red boxes indicate cropped regions shown in the main figure. GAPDH and β-actin were used as loading controls.


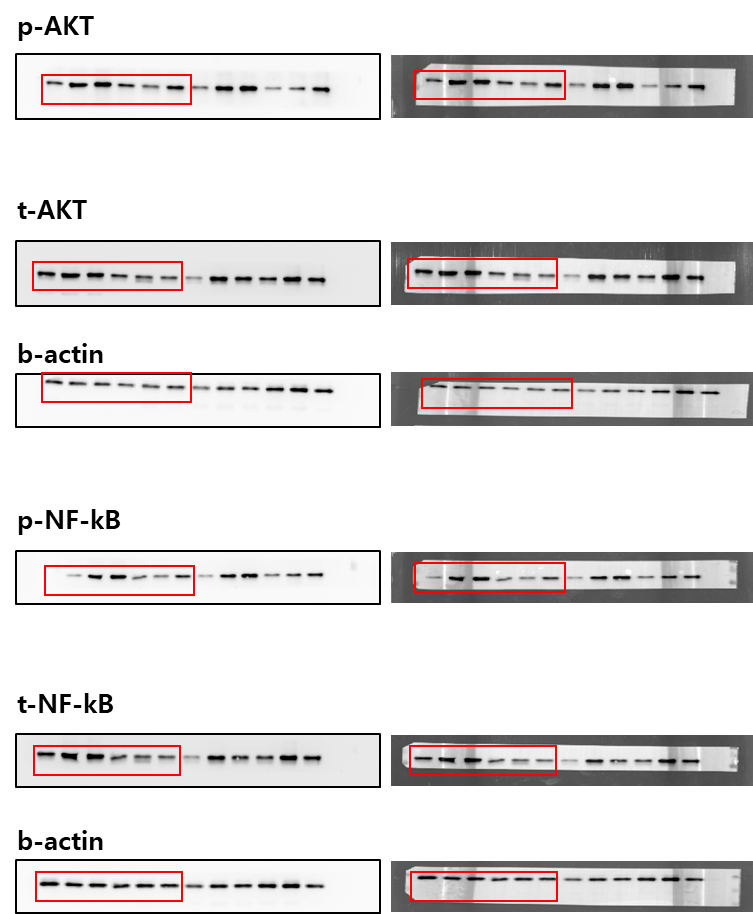


Supplementary Figure S5. Uncropped full-length Western blot membranes corresponding to Figure 5 (p-AKT, t-AKT, p-NF-κB, t-NF-κB). Red boxes indicate cropped regions shown in the main figure. β-actin was used as a loading control.
